# Retraction statement: Ding, J., Wang, X., Gao, J. and Song, T. (2021), Silencing of cystatin SN abrogates cancer progression and stem cell properties in papillary thyroid carcinoma. FEBS Open Bio, 11: 2186–2197.

**DOI:** 10.1002/2211-5463.13664

**Published:** 2023-07-06

## Abstract

https://doi.org/10.1002/2211-5463.13221

The above article, published online on 08 June 2021 in Wiley Online Library (wileyonlinelibrary.com), has been retracted by agreement between the authors, the Editor‐in‐Chief Miguel De la Rosa, FEBS Press, and John Wiley and Sons Ltd. The retraction has been agreed following an investigation into concerns raised by a third party, which revealed inappropriate duplications between this and articles that were either previously published or published later in the same year [1–9]. Thus, the editors consider the conclusions of this manuscript substantially compromised.

[1] Zheng, X., Huang, M., Xing, L. et al. The circRNA circSEPT9 mediated by E2F1 and EIF4A3 facilitates the carcinogenesis and development of triple‐negative breast cancer. Mol Cancer 19, 73 (2020). https://doi.org/10.1186/s12943-020-01183-9.

[2] Li X, Wang H, Liu Z, Abudureyimu A. CircSETD3 (Hsa_circ_0000567) Suppresses Hepatoblastoma Pathogenesis via Targeting the miR‐423‐3p/Bcl‐2‐Interacting Mediator of Cell Death Axis. Front Genet. 2021 Sep 29;12:724197. doi: 10.3389/fgene.2021.724197. PMID: 34659347; PMCID: PMC8511783.

[3] Du J, Zhong H, Ma B. Targeting a novel LncRNA SNHG15/miR‐451/c‐Myc signaling cascade is effective to hamper the pathogenesis of breast cancer (BC) in vitro and in vivo. Cancer Cell Int. 2021 Mar 31;21(1):186. doi: 10.1186/s12935‐021‐01885‐0. PMID: 33952250; PMCID: PMC8097789.

[4] Hong W, Xue M, Jiang J, Zhang Y, Gao X. Circular RNA circ‐CPA4/ let‐7 miRNA/PD‐L1 axis regulates cell growth, stemness, drug resistance and immune evasion in non‐small cell lung cancer (NSCLC). J Exp Clin Cancer Res. 2020 Aug 3;39(1):149. doi: 10.1186/s13046‐020‐01648‐1. PMID: 32746878; PMCID: PMC7397626.

[5] Ren N, Jiang T, Wang C, Xie S, Xing Y, Piao D, Zhang T, Zhu Y. LncRNA ADAMTS9‐AS2 inhibits gastric cancer (GC) development and sensitizes chemoresistant GC cells to cisplatin by regulating miR‐223‐3p/NLRP3 axis. Aging (Albany NY). 2020 Jun 9;12(11):11025–11,041. doi: 10.18632/aging.103314. Epub 2020 Jun 9. PMID: 32516127; PMCID: PMC7346038.

[6] Zheng Y, Liu L, Wang Y, Xiao S, Mai R, Zhu Z, Cao Y. Glioblastoma stem cell (GSC)‐derived PD‐L1‐containing exosomes activates AMPK/ULK1 pathway mediated autophagy to increase temozolomide‐resistance in glioblastoma. Cell Biosci. 2021 Mar 31;11(1):63. doi: 10.1186/s13578‐021‐00575‐8. PMID: 33789726; PMCID: PMC8011168.

[7] Lin H, Wang J, Wang T, Wu J, Wang P, Huo X, Zhang J, Pan H, Fan Y. The LncRNA MIR503HG/miR‐224‐5p/TUSC3 Signaling Cascade Suppresses Gastric Cancer Development via Modulating ATF6 Branch of Unfolded Protein Response. Front Oncol. 2021 Jul 26;11:708501. doi: 10.3389/fonc.2021.708501. PMID: 34381729; PMCID: PMC8352579.

[8] Lu G, Li Y, Ma Y, Lu J, Chen Y, Jiang Q, Qin Q, Zhao L, Huang Q, Luo Z, Huang S, Wei Z. Long noncoding RNA LINC00511 contributes to breast cancer tumourigenesis and stemness by inducing the miR‐185‐3p/E2F1/Nanog axis. J Exp Clin Cancer Res. 2018 Nov 27;37(1):289. doi: 10.1186/s13046‐018‐0945‐6. PMID: 30482236; PMCID: PMC6260744.

[9] Zhao Y, Zheng R, Chen J, Ning D. CircRNA CDR1as/miR‐641/HOXA9 pathway regulated stemness contributes to cisplatin resistance in non‐small cell lung cancer (NSCLC). Cancer Cell Int. 2020 Jul 6;20:289. doi: 10.1186/s12935‐020‐01390‐w. PMID: 32655321; PMCID: PMC7339514.

https://doi.org/10.1002/2211-5463.13221

The above article, published online on 08 June 2021 in Wiley Online Library (wileyonlinelibrary.com), has been retracted by agreement between the authors, the Editor‐in‐Chief Miguel De la Rosa, FEBS Press, and John Wiley and Sons Ltd. The retraction has been agreed following an investigation into concerns raised by a third party, which revealed inappropriate duplications between this and articles that were either previously published or published later in the same year [1–9]. Thus, the editors consider the conclusions of this manuscript substantially compromised.

[1] Zheng, X., Huang, M., Xing, L. et al. The circRNA circSEPT9 mediated by E2F1 and EIF4A3 facilitates the carcinogenesis and development of triple‐negative breast cancer. Mol Cancer 19, 73 (2020). https://doi.org/10.1186/s12943-020-01183-9.

[2] Li X, Wang H, Liu Z, Abudureyimu A. CircSETD3 (Hsa_circ_0000567) Suppresses Hepatoblastoma Pathogenesis via Targeting the miR‐423‐3p/Bcl‐2‐Interacting Mediator of Cell Death Axis. Front Genet. 2021 Sep 29;12:724197. doi: 10.3389/fgene.2021.724197. PMID: 34659347; PMCID: PMC8511783.

[3] Du J, Zhong H, Ma B. Targeting a novel LncRNA SNHG15/miR‐451/c‐Myc signaling cascade is effective to hamper the pathogenesis of breast cancer (BC) in vitro and in vivo. Cancer Cell Int. 2021 Mar 31;21(1):186. doi: 10.1186/s12935‐021‐01885‐0. PMID: 33952250; PMCID: PMC8097789.

[4] Hong W, Xue M, Jiang J, Zhang Y, Gao X. Circular RNA circ‐CPA4/ let‐7 miRNA/PD‐L1 axis regulates cell growth, stemness, drug resistance and immune evasion in non‐small cell lung cancer (NSCLC). J Exp Clin Cancer Res. 2020 Aug 3;39(1):149. doi: 10.1186/s13046‐020‐01648‐1. PMID: 32746878; PMCID: PMC7397626.

[5] Ren N, Jiang T, Wang C, Xie S, Xing Y, Piao D, Zhang T, Zhu Y. LncRNA ADAMTS9‐AS2 inhibits gastric cancer (GC) development and sensitizes chemoresistant GC cells to cisplatin by regulating miR‐223‐3p/NLRP3 axis. Aging (Albany NY). 2020 Jun 9;12(11):11025–11,041. doi: 10.18632/aging.103314. Epub 2020 Jun 9. PMID: 32516127; PMCID: PMC7346038.

[6] Zheng Y, Liu L, Wang Y, Xiao S, Mai R, Zhu Z, Cao Y. Glioblastoma stem cell (GSC)‐derived PD‐L1‐containing exosomes activates AMPK/ULK1 pathway mediated autophagy to increase temozolomide‐resistance in glioblastoma. Cell Biosci. 2021 Mar 31;11(1):63. doi: 10.1186/s13578‐021‐00575‐8. PMID: 33789726; PMCID: PMC8011168.

[7] Lin H, Wang J, Wang T, Wu J, Wang P, Huo X, Zhang J, Pan H, Fan Y. The LncRNA MIR503HG/miR‐224‐5p/TUSC3 Signaling Cascade Suppresses Gastric Cancer Development via Modulating ATF6 Branch of Unfolded Protein Response. Front Oncol. 2021 Jul 26;11:708501. doi: 10.3389/fonc.2021.708501. PMID: 34381729; PMCID: PMC8352579.

[8] Lu G, Li Y, Ma Y, Lu J, Chen Y, Jiang Q, Qin Q, Zhao L, Huang Q, Luo Z, Huang S, Wei Z. Long noncoding RNA LINC00511 contributes to breast cancer tumourigenesis and stemness by inducing the miR‐185‐3p/E2F1/Nanog axis. J Exp Clin Cancer Res. 2018 Nov 27;37(1):289. doi: 10.1186/s13046‐018‐0945‐6. PMID: 30482236; PMCID: PMC6260744.

[9] Zhao Y, Zheng R, Chen J, Ning D. CircRNA CDR1as/miR‐641/HOXA9 pathway regulated stemness contributes to cisplatin resistance in non‐small cell lung cancer (NSCLC). Cancer Cell Int. 2020 Jul 6;20:289. doi: 10.1186/s12935‐020‐01390‐w. PMID: 32655321; PMCID: PMC7339514.

